# Exploring the Nature of Silicon-Noble Gas Bonds in H_3_SiNgNSi and HSiNgNSi Compounds (Ng = Xe, Rn)

**DOI:** 10.3390/ijms16036402

**Published:** 2015-03-19

**Authors:** Sudip Pan, Ranajit Saha, Pratim K. Chattaraj

**Affiliations:** Department of Chemistry and Centre for Theoretical Studies, Indian Institute of Technology, Kharagpur 721302, India; E-Mails: sudip.chem88@gmail.com (S.P.); ranajitsahachem@gmail.com (R.S.)

**Keywords:** *ab initio* study, dissociation channels, kinetic stability, natural population analysis, electron density analysis, energy decomposition analysis

## Abstract

*Ab initio* and density functional theory-based computations are performed to investigate the structure and stability of H_3_SiNgNSi and HSiNgNSi compounds (Ng = Xe, Rn). They are thermochemically unstable with respect to the dissociation channel producing Ng and H_3_SiNSi or HSiNSi. However, they are kinetically stable with respect to this dissociation channel having activation free energy barriers of 19.3 and 23.3 kcal/mol for H_3_SiXeNSi and H_3_SiRnNSi, respectively, and 9.2 and 12.8 kcal/mol for HSiXeNSi and HSiRnNSi, respectively. The rest of the possible dissociation channels are endergonic in nature at room temperature for Rn analogues. However, one three-body dissociation channel for H_3_SiXeNSi and one two-body and one three-body dissociation channels for HSiXeNSi are slightly exergonic in nature at room temperature. They become endergonic at slightly lower temperature. The nature of bonding between Ng and Si/N is analyzed by natural bond order, electron density and energy decomposition analyses. Natural population analysis indicates that they could be best represented as (H_3_SiNg)^+^(NSi)^−^ and (HSiNg)^+^(NSi)^−^. Energy decomposition analysis further reveals that the contribution from the orbital term (ΔE_orb_) is dominant (*ca*. 67%–75%) towards the total attraction energy associated with the Si-Ng bond, whereas the electrostatic term (ΔE_elstat_) contributes the maximum (*ca*. 66%–68%) for the same in the Ng–N bond, implying the covalent nature of the former bond and the ionic nature of the latter.

## 1. Introduction

Having a late break-through in 1962 with the discovery of Xe^+^[PtF_6_]^−^, chemistry related to the noble gas (Ng) compounds has been developing very rapidly, especially during the last two decades. This is due to the outcome of the huge efforts made by both experimentalists [1,2,3,4,5,6,7,8,9,10,11,12,13,14,15,16,17,18,19] and theoreticians [20,21,22,23,24,25,26,27,28,29,30,31,32,33,34,35,36,37,38,39,40,41,42,43,44,45,46] towards the syntheses and/or predictions of stable Ng-containing compounds. The first compound having the Xe–C bond was reported as XeCH_3_^+^ many years ago in 1961 by Field *et al.* [47], and the very next year, the same group [48] detected ArCH_3_^+^ and KrCH_3_^+^ in mass spectrum. A few years later in 1979, Turbini *et al.* [49] detected Xe(CF_3_)_2_ by means of mass spectroscopic techniques. Subsequently, detection of a few pentafluorophenylxenon cation [50,51,52] derivatives was also reported in 1989. Thereafter, plenty of examples with Ng–C bonds, *viz*. HXeCCH [53,54,55], HKrCCH [55], HXeCC [53,55], HXeCCXeH [53,55], HXeCN [5], HKrCN [5], HNgCCF [56], HCCNgF [56], ClXeCN [57], BrXeCN [57], HXeC_3_N [58], HKrC_3_N [58], HXeC_4_H [11] and HKrC_4_H [11], were made available in the literature as experimentally-detected compounds using a low temperature matrix-isolation technique. Moreover, several theoretically-predicted compounds with Ng–C bonds, some unusual highly-coordinated Ng(CCH)_4_ and Ng(CCH)_6_ clusters (Ng = Kr, Xe) [59] and polymer, H–(Xe–C_2_)_n_–Xe–H (*n* ≥ 1) [60], were found to be metastable species.

On the other hand, there are only a few examples of the systems having Si-Ng bonds, which were experimentally obtained or theoretically predicted to be viable. The generation of the F_3_SiXe^+^ by Grandinetti *et al.* [61] in an ion-molecule reaction between the protonated SiF_4_ and Xe, F_3_Si–FH^+^ + Xe → Xe–SiF_3_^+^ + HF, provided the first system with the Si–Ng bond. Through so-called direct addition of SiF_3_^+^ and Ng, Cunje *et al.* [62] were successful in producing not only XeSiF_3_^+^, but also ArSiF_3_^+^ and KrSiF_3_^+^ at room temperature and high pressure. Two other isomers of NgSiF_3_^+^ with Si–Ng–F and F–Ng–F types of linkages were also proposed [62]. The metastability of the first neutral compound, FArSiF_3_, with the Si–Ar chemical bond, was predicted by Cohen *et al.* [63]. Prompted by this study, Yockel *et al.* [64] found viable FKrSiF_3_ as the first example having the Si–Kr bond in a neutral system. Lundell and coworkers [65] further assessed the stability and bonding of FXeSiF, the first neutral compound with the Si–Xe covalent bond. Roithová *et al.* [66] showed that SiF_3_^2+^ could behave as a superelectrophilic reagent and that NgSiF_2_^2+^ (Ng = Ne, Ar) could be formed as a result of the thermal ion-molecule reaction, F_3_Si^2+^ + Ng → NgSiF_2_^2+^ + F. Recently, Savoca *et al.* [67] detected Si_4_Xe^+^ via infrared (IR) multiple photon dissociation spectroscopy. More recently, we studied the Ng binding ability of the SiH_3_^+^ cluster, as well as the effect of H substitution of SiH_3_^+^ by halide groups (–X) on its ability in binding Ng [68].

In this manuscript, we have reported two new viable compounds, H_3_SiNgNSi and HSiNgNSi (Ng = Xe, Rn), with Si-Ng covalent bonds. Crabtree and coworkers [69] very recently detected highly stable silicon nitrides, H_3_SiNSi and HSiNSi by chirped-pulse Fourier transform microwave (CP-FTMW) spectroscopy. H_3_SiNSi has a C_3v_ point group symmetry with a linear Si–N–Si moiety, whereas HSiNSi possesses a planar geometry (C_s_) with a slightly bent Si–N–Si arrangement, and for both of them, the minimum energy structures have a singlet spin state. We have assessed *in silico* the structure, stability and the nature of bonding in H_3_SiNgNSi and HSiNgNSi compounds. They are found to be metastable systems. The nature of bonding therein is analyzed by natural population analysis (NPA), Wiberg bond indices (WBI) calculation [70], electron density analysis [71] and energy decomposition analysis (EDA) [72,73,74,75]. It may be noted that except for RnSiX_3_^+^ (X = H, F–Br) [68], there is no study with the compound having the Si-Rn bond. In that sense, for the first time, we reported here the neutral Rn-containing compounds, H_3_SiRnNSi and HSiRnNSi, with the Si–Rn covalent bond.

## 2. Results and Discussion

### 2.1. Structure and Stability

**Figure 1:**
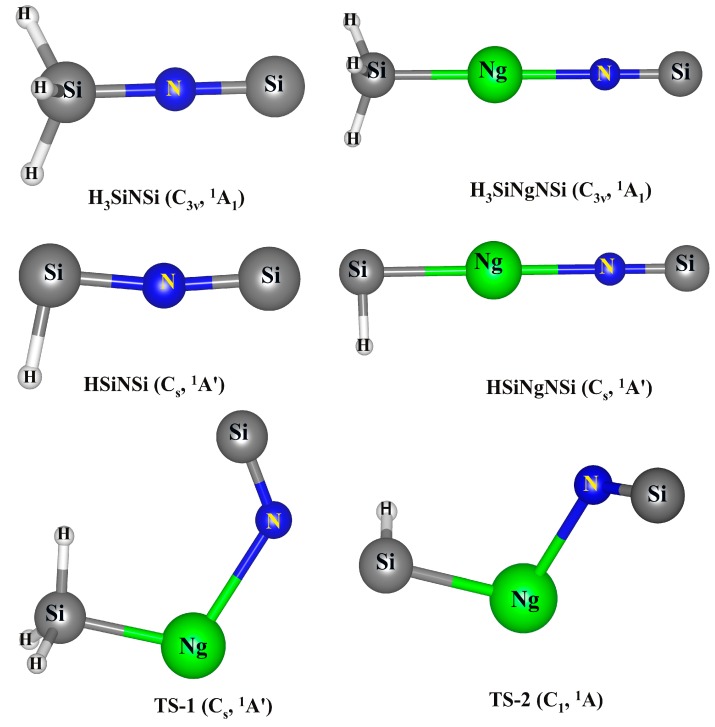
Pictorial depictions of the energy minimum structures and the transition states (TSs) of H_3_SiNSi, HSiNSi, H_3_SiNgNSi and HSiNgNSi compounds. Point groups along with their electronic states are given in parentheses. **TS-1** and **TS-2** are associated with the dissociation of H_3_SiNgNSi and HSiNgNSi, producing Ng and H_3_SiNSi or HSiNSi.

The optimized geometries of H_3_SiNSi and HSiNSi compounds and their Ng inserted analogues, H_3_SiNgNSi and HSiNgNSi, are provided in Figure 1. Similar to their mother moieties, the minimum energy structure of H_3_SiNgNSi corresponds to the *C*_3v_ point group with the ^1^A_1_ electronic state, whereas HSiNgNSi has a planar geometry with the *C*_s_ point group and ^1^A' electronic state. On the other hand, the transition states (TSs) corresponding to the dissociations of H_3_SiNgNSi and HSiNgNSi into Ng and H_3_SiNSi or HSiNSi have *C*_s_ (**TS-1** in Figure 1) and *C*_1_ (**TS-2** in Figure 1) symmetry, respectively, in which the NSi fragment remains attached with H_3_SiNg or HSiNg fragments in a tilted fashion. The geometrical parameters of H_3_SiNgNSi and HSiNgNSi compounds obtained at the ωB97X-D/def2-QZVPPD and CCSD(T)/def2-TZVP levels are provided in Table S1 (Supplementary Information), whereas the same for the minimum energy and TS structures of H_3_SiNgNSi and HSiNgNSi obtained at the MP2/def2-QZVPPD level are given in Table 1. The geometrical parameters of H_3_SiNSi and HSiNSi are also provided in Table S2 (Supplementary Information). The Si–Ng bond distance in H_3_SiNgNSi is somewhat shorter than that in HSiNgNSi. This may be due to the positive charges on the Ng centers in H_3_SiNgNSi and HSiNgNSi compounds. The positive charge on the Ng center in H_3_SiNgNSi (+0.61 |*e*| on Xe and +0.68 |*e*| on Rn) is larger than that in HSiNgNSi (+0.50 |*e*| on Xe and +0.57 |*e*| on Rn). The chemical inertness of Ng atoms originates from the filled valence shell. Hence, a more positively-charged Ng center would be more effective in taking part in chemical bond formation. The larger interaction energy between H_3_Si and NgNSi than that between HSi and NgNSi corroborates well with the stronger bond formation in the former cases than the latter ones (*vide infra*).

The N–Si bond gets slightly elongated in the Ng inserted analogues compared to those in H_3_SiNSi and HSiNSi. The Si–Ng–N and Ng–N–Si moieties in the H_3_SiNgNSi compound are linear. However, the same in the HSiNgNSi compound are slightly bent from the linear arrangement (≤0.5°). Since in **TS-1** and **TS-2**, the NSi fragment is bonded to the Ng center in a tilted fashion, having the mode with imaginary frequency as the bending of Si–Ng–N and Ng–N–Si angles, the <Si–Ng–N and <Ng–N–Si get shortened (96.0°–126.2°) compared to those in the corresponding minimum energy structures (~180.0°). In the TSs, the Si–Ng bond distance is found to decrease by about 0.1 Å, while the Ng–N bond distance increases by 0.3–0.2 Å compared to those in the corresponding minimum energy structures.

**Table 1:** The geometrical parameters (in Å and degrees) of the optimized geometries of H_3_SiNgNSi and HSiNgNSi compounds (both minimum energy structures and transition states) studied at the MP2/def2-QZVPPD level.

Nature of Stationary Points	Compounds	r_H–Si_	r_Si–Ng_	r_Ng–N_	r_N–Si_	<H–Si–Ng	<Si–Ng–N	<Ng–N–Si
Minimum	H_3_SiXeNSi	1.471	2.588	2.338	1.600	107.0	180.0	180.0
Energy	H_3_SiRnNSi	1.472	2.688	2.382	1.598	107.8	180.0	180.0
Structures	HSiXeNSi	1.508	2.653	2.375	1.603	88.7	179.5	178.6
	HSiRnNSi	1.510	2.747	2.420	1.601	89.0	179.7	179.9
Transition	H_3_SiXeNSi	1.462(3)	2.486	2.683	1.616	103.9, 99.5	110.6	126.2
States	H_3_SiRnNSi	1.463(5)	2.573	2.733	1.616	104.1, 99.3	103.7	125.9
	HSiXeNSi	1.504	2.511	2.576	1.626	87.6	100.7	125.6
	HSiRnNSi	1.505	2.590	2.623	1.625	87.7	96.0	125.5

The stability of these Ng inserted compounds is understood by computing ZPE-corrected dissociation energy (D_0_), as well as dissociation enthalpy (ΔH) and free energy change (ΔG) at 298 K for different possible dissociation channels. We have considered the higher spin states of all of the dissociated products. The spin state, which gives the lowest energy, is taken into consideration. We have computed ΔG values for the different possible dissociation channels of H_3_SiNgNSi and HSiNgNSi at both the MP2/def2-QZVPPD (see Table S3 in the Supplementary Information) and ωB97X-D/def2-QZVPPD levels (see Table 2), and those for the D_0_ and ΔH are given in Tables S4 and S5 (Supplementary Information). We found that the D_0_, ΔH and ΔG values obtained at the MP2 level are larger than those obtained at the ωB97X-D level in most cases, particularly in Xe analogues. In some cases, this provides qualitatively wrong results. For example, with respect to most of the dissociation channels, the Xe inserted compounds are less likely to be dissociated than those of the Rn analogues, implying larger stability of the former compounds than the latter ones. However, in general, it is expected that due to larger polarizability, Rn would make a somewhat stronger bond than that of Xe. It was already reported in the literature that the MP2 level of computation can produce inaccurate dissociation energy diagrams for the Ng inserted compounds [76]. Our results corroborate that the stability of Ng inserted compounds should not be analyzed based on MP2 results alone. Therefore, we have given special emphasis to the results obtained at the ωB97X-D level to assess the stability of these studied compounds with respect to the different dissociation channels.

We have considered two-body (2-B) as well as three-body (3-B) dissociation channels comprising both neutral and ionic fragments. For H_3_SiNgNSi, except for the 2-B dissociation channel producing H_3_SiNSi and Ng, all other dissociation channels are endergonic in nature at room temperature. Though dissociation of H_3_SiNgNSi into H_3_SiNSi, and Ng is exergonic by −119.3 kcal/mol for Xe and −110.5 kcal/mol for Rn, the dissociation is kinetically protected by 19.3 and 23.3 kcal/mol for Xe and Rn analogues, respectively. For H_3_SiXeNSi, except this 2-B dissociation, another 3-B dissociation producing H_3_Si, Ng and NSi is slightly exergonic in nature (−0.2 kcal/mol) at room temperature. However, it becomes endergonic (2.6 kcal/mol) at a slightly lower temperature (250 K), as the contribution from the favorable ΔS term becomes smaller at a lower temperature.

In the case of HSiRnNSi, except for the dissociation into HSiNSi and Rn, all other dissociation channels are endergonic in nature. However, for HSiXeNSi, in addition to the dissociation into HSiNSi and Xe, two other 2-B and 3-B dissociations are slightly exergonic in nature at 298 K. In both Xe and Rn analogues, the dissociation producing HSiNSi and Ng is highly exergonic, being −121.1 kcal/mol for Xe and −113.0 kcal/mol for Rn. This dissociation is found to be kinetically protected by the free energy barrier of 9.2 kcal/mol for Xe and 12.8 kcal/mol for Rn analogues. The 2-B dissociation of HSiXeNSi producing HSiXe and NSi and the 3-B dissociation producing HSi, Xe and NSi are exergonic by −3.6 and −5.8 kcal/mol, respectively, at 298 K. We have computed ΔG values at a lower temperature and have found that at 180 K, ΔG values become slightly positive (0.5 and 0.2 kcal/mol for 2-B and 3-B dissociations, respectively), and at 150 K, it becomes 1.6 kcal/mol for the 2-B dissociation and 1.7 kcal/mol for the 3-B dissociation. It may be noted that activation free energy barriers obtained at the MP2 level are quite close to those obtained at the ωB97X-D level (see Table S3).

**Table 2:** Free energy change (ΔG, kcal/mol) at 298 K for different dissociation channels of H_3_SiNgNSi and HSiNgNSi compounds at the ωB97X-D/def2-QZVPPD level.

Processes	ΔG		Processes	ΔG	
	Xe	Rn		Xe	Rn
H_3_SiNgNSi → H_3_SiNg^+^ + NSi^−^	105.3	110.7	HSiNgNSi → HSiNg^+^ + NSi^−^	99.1	103.8
H_3_SiNgNSi → H_3_Si^−^ + NgNSi^+^	165.2	166.1	HSiNgNSi → HSiNg + NSi	−3.6	3.8
H_3_SiNgNSi → H_3_SiNSi + Ng	−119.3	−110.5	HSiNgNSi → HSi^−^ + NgNSi^+^	163.6	163.9
H_3_SiNgNSi → H_3_Si + Ng + NSi	−0.2	8.6	HSiNgNSi → HSiNSi + Ng	−121.1	−113.0
H_3_SiNgNSi → H_3_Si^+^ + Ng + NSi^−^	122.0	130.8	HSiNgNSi → HSi + Ng + NSi	−5.8	2.4
H_3_SiNgNSi → H_3_Si^−^ + Ng + NSi^+^	201.6	210.4	HSiNgNSi → HSi^+^ + Ng + NSi^−^	112.9	121.1
H_3_SiNgNSi → H_2_Si + NgH + NSi	64.4	73.0	HSiNgNSi → HSi^−^ + Ng + NSi^+^	199.9	208.1
H_3_SiNgNSi → H_2_Si^+^ + NgH + NSi^−^	206.6	215.2	HSiNgNSi → Si + NgH + NSi	87.7	95.7
H_3_SiNgNSi → H_2_Si^−^ + NgH + NSi^+^	271.9	280.6	HSiNgNSi → Si + NgH^+^ + NSi^−^	220.2	221.9
H_3_SiNgNSi → H_2_Si + NgH^+^ + NSi^−^	196.9	199.2	ΔG^‡a^	9.2	12.8
H_3_SiNgNSi → H_2_Si^−^ + NgH^+^ + NSi	236.9	239.2	−	−	−
H_3_SiNgNSi → HSi + HNgH + NSi	149.4	149.8	−	−	−
H_3_SiNgNSi → HSi^+^ + HNgH + NSi^−^	268.1	268.4	−	−	−
H_3_SiNgNSi → HSi^−^ + HNgH + NSi^+^	355.1	355.5	−	−	−
ΔG^‡a^	19.3	23.3	−	−	−

ΔG^‡a^ is the activation free energy barrier for the processes, H_3_SiNgNSi → H_3_SiNSi + Ng and HSiNgNSi → HSiNSi + Ng.

Hu and co-workers [77] argued that to have a half-life in the order of ~10^2^ s at 100, 200 and 300 K, a system of type XNgY must have a minimum energy barrier of 6, 13 and 21 kcal/mol, respectively. Therefore, H_3_SiNgNSi could be detected at as high as a 250–300 K temperature range, whereas HSiNgNSi could be detected around the 150–200 K temperature range.

### 2.2. Nature of Bonding

The NPA charge at each atomic center and the WBI values of Si–Ng and Ng–N bonds are tabulated in Table 3. The H and N centers are electronegative in nature, while Si and Ng centers are electropositive in nature. The Si center attached to N (0.77–0.80 |*e*|) carries a slightly more positive charge than that of Si in the –SiH_3_ fragment (0.51–0.63 |*e*|). On the other hand, N attains a large negative charge of −1.50 |*e|* for Xe analogues and −1.52(4) |*e*| for Rn analogues. Note that the net charge on the NSi fragment ranges from −0.71 |*e*| to −0.75 |*e|*. Therefore, they could be best represented as (H_3_SiNg)^+^(NSi)^−^ and (HSiNg)^+^(NSi)^−^. Obviously, the Ng–N bond would be of the ionic type. The low WBI values (~0.2) for the Ng–N bonds dictate their ionic nature of interaction. In contrast, the quite high WBI values (~0.65) for the Si–Ng bonds imply that the bonds are of the covalent type, and almost a single bond is formed therein.

**Table 3:** Natural population analysis (NPA) charge on each atomic center (q_k_, au) and Wiberg bond indices (WBI) values of Si–Ng and Ng–N bonds computed at the MP2/def2-QZVPPD level.

Compounds	q_k_					WBI	
	H	Si	Ng	N	Si	Si–Ng	Ng–N
H_3_SiXeNSi	−0.17	+0.63	+0.61	−1.50	+0.79	0.64	0.22
H_3_SiRnNSi	−0.17	+0.56	+0.68	−1.52	+0.80	0.65	0.22
HSiXeNSi	−0.33	+0.56	+0.50	−1.50	+0.77	0.63	0.18
HSiRnNSi	−0.33	+0.51	+0.57	−1.54	+0.79	0.66	0.17

Electron density analysis [71] provides additional insight into the nature of bonding. Different topological descriptors of electron density and electron localization function (ELF) computed at the bond critical points (BCPs) of Si–Ng and Ng–N bonds are given in Table 4. The concentration and depletion of electron density at the BCPs are indicated by the negative and positive values of ∇^2^ρ(**r_c_**), respectively. In general, the occurrence of electron density concentration and depletion at the BCPs indicates the covalent and noncovalent type of bonding, respectively. However, many failures ([71,78,79,80,81,82,83] pp. 312–314) of this descriptor in representing a covalent bond, especially for the systems with heavy atoms, are documented in the literature. The local electron energy density (H(**r_c_**)), which is the sum of local kinetic energy density (G(**r_c_**)) and local potential energy density (V(**r_c_**)), is also commonly applied to interpret the nature of a bond.

**Table 4:** Calculated topological properties (au) at the bond critical points of Ng–Si and Ng–N bonds obtained from the .wfn files generated at the MP2/def2-QZVPPD level.

Compounds	ρ(r_c_)	∇^2^ρ(r_c_)	G(r_c_)	V(r_c_)	H(r_c_)	ELF
H_3_Si^__^●^__^XeNSi	0.078	−0.093	0.016	−0.055	−0.039	0.868
H_3_SiXe^__^●^__^NSi	0.073	0.140	0.057	−0.079	−0.022	0.295
H_3_Si^__^●^__^RnNSi	0.075	−0.061	0.018	−0.051	−0.033	0.824
H_3_SiRn^__^●^__^NSi	0.073	0.139	0.058	−0.081	−0.023	0.284
HSi^__^●^__^XeNSi	0.069	−0.056	0.017	−0.049	−0.032	0.786
HSiXe^__^●^__^NSi	0.068	0.137	0.053	−0.072	−0.019	0.278
HSi^__^●^__^RnNSi	0.066	−0.042	0.017	−0.045	−0.028	0.769
HSiRn^__^●^__^NSi	0.068	0.132	0.053	−0.073	−0.020	0.276

**Figure 2:**
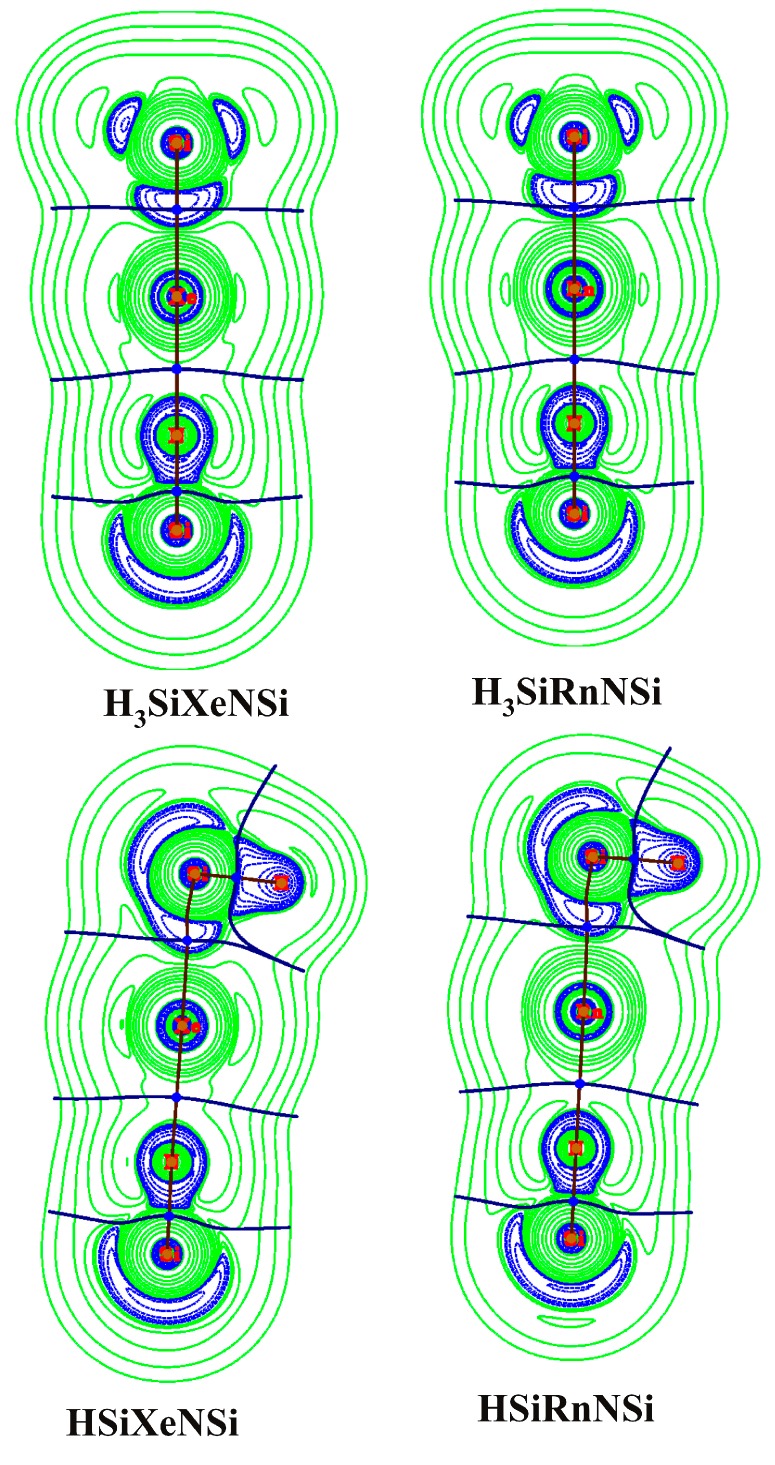
Contour plots of the Laplacian of the electron density of H_3_SiXeNSi and HSiXeNSi clusters at a particular plane computed at the MP2/def2-QZVPPD/WTBS level (WTBS is used for Xe and Rn; The green-colored region shows the area of ∇^2^ρ(**r**) > 0, whereas the blue-colored region shows the area of ∇^2^ρ(**r**) < 0).

Even if ∇^2^ρ(**r_c_**) > 0, but H(**r_c_**) < 0, then also the bond might be considered as a covalent type [84]. In our cases, ∇^2^ρ(**r_c_**) is negative in the Si–Ng bonds, implying their covalent nature. However, H(**r_c_**) is negative in both Si–Ng and Ng–N bonds and slightly more negative in Si–Ng bonds than that in Ng–N bonds. The contour plots of ∇^2^ρ(**r**) are displayed in Figure 2.

**Figure 3:**
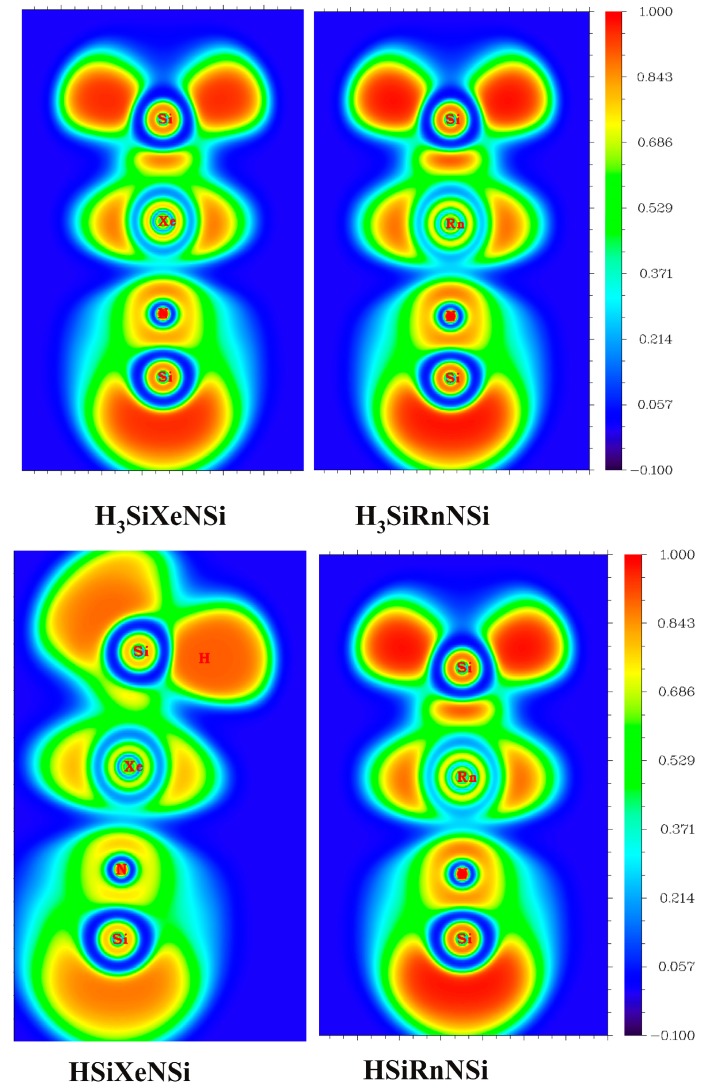
Color-filled maps of the electron localization function of H_3_SiXeNSi and HSiXeNSi clusters at a particular plane computed at the MP2/def2-QZVPPD/WTBS level (WTBS is used for Xe and Rn).

In between Si and Ng centers, a well-defined region having ∇^2^ρ(**r**) < 0 is developed, whereas in between Ng and N centers, the valence orbitals only undergo slight deformation in their shapes. Note that though H(**r_c_**) is negative in Ng–N bonds, the charge distribution shows that it would be better to consider them as ionic bonds rather than the covalent ones. To prove this, we have further computed ELF [85] at the BCPs of Si–Ng and Ng–N bonds, and the corresponding color-filled maps of ELF are provided in Figure 3. Generally, a high value of ELF at a certain point is an indicator of the localized electrons therein. It further implies the existence of covalent bonds or lone pairs or core electrons.

A typical covalent bond possesses a large ELF value in between two bonded centers, whereas in the case of an ionic bond, the ELF value at the interstitial positions of the two atoms is very low. In our cases, the ELF values at the BCPs of Si–Ng bonds are quite high (~0.8) being close to the limiting value of 1.0 for a perfect localization case, whereas they are quite small (~0.3) for the Ng–N cases, corroborating well with their ionic nature. The color-filled maps of ELF further dictate the large degree of electron localization in between Si and Ng centers, whereas it is very small in between Ng and N centers (see Figure 3).

The total interaction energy (ΔE_int_) is divided into the Pauli repulsion (ΔE_pauli_), electrostatic (E_elstat_), orbital (ΔE_orb_) and dispersion (ΔE_disp_) energy terms in EDA to get further insight into the nature of Si–Ng and Ng–N bonds (see Table 5). The NPA charge on each atomic center is used as a guiding tool to impose the charges on the fragments used in our energy partitioning schemes. Since H_3_SiNgNSi and HSiNgNSi could be best represented as (H_3_SiNg)^+^(NSi)^−^ and (HSiNg)^+^(NSi)^−^, we have used [H_3_SiNg]^+^ or [HSiNg]^+^ and [NSi]^−^ as two fragments to know the nature of the Ng–N bond. As Tonner and Frenking [86] argued, when two different fragmentation schemes are possible, the most favorable one is given by the smallest size of the ΔE_orb_ term. Therefore, we also performed EDA following the radical fragmentation scheme, and indeed, the ionic fragmentation gives a smaller ΔE_orb_ value than that of the radical one. On the other hand, since the total charges on the fragments, [H_3_Si] or [HSi] and [NgNSi], are well below 0.5 |*e|*, we have partitioned H_3_SiNgNSi and HSiNgNSi into neutral [H_3_Si] or [HSi] and [NgNSi] fragments to explore the nature of Si–Ng bond, as the total charges on them are below 0.5 |*e*|.

**Table 5:** Energy decomposition analysis (EDA) results of the H_3_SiNgNSi and HSiNgNSi molecules studied at the revPBE-D3/TZ2P//MP2/def2-QZVPPD level. All of the energy terms are in kcal/mol.

Compounds	Fragments	ΔE_int_	ΔE_pauli_	ΔE_elstat_	ΔE_orb_	ΔE_disp_
H_3_SiXeNSi	[H_3_Si] + [XeNSi]	−46.0	210.4	−82.0 (32.0%)	−172.6 (67.3%)	−1.7 (0.7%)
	[H_3_SiXe]^+^ + [NSi]^−^	−128.1	111.6	−159.4 (66.5%)	−78.6 (32.8%)	−1.7 (0.7%)
H_3_SiRnNSi	[H_3_Si] + [RnNSi]	−49.9	198.8	−80.0 (32.2%)	−166.9 (67.1%)	−1.8 (0.7%)
	[H_3_SiRn]^+^ + [NSi]^−^	−132.9	113.0	−166.6 (67.7%)	−77.4 (31.5%)	−1.9 (0.8%)
HSiXeNSi	[HSi] + [XeNSi]	−37.8	169.6	−51.0 (24.6%)	−155.4 (74.9%)	−1.0 (0.5%)
	[HSiXe]^+^ + [NSi]^−^	−120.4	103.4	−148.0 (66.2%)	−74.0 (33.1%)	−1.7 (0.8%)
HSiRnNSi	[HSi] + [RnNSi]	−41.0	163.9	−50.9 (24.8%)	−153.0 (74.7%)	−1.1 (0.5%)
	[HSiRn]^+^ + [NSi]^−^	−123.7	105.3	−154.4 (67.4%)	−72.7 (31.7%)	−1.9 (0.8%)

(The percentage values within the parentheses show the contribution towards the total attractive interaction ΔE_elstat_ + ΔE_orb_ + ΔE_disp_).

In the Ng–N bonds, the contribution from ΔE_elstat_ towards the total attraction is the maximum ranging within 66%–68%. ΔE_orb_ contributes around 31%–33% towards the total attraction in these bonds. In cases of Si–Ng bonds, ΔE_orb_ is the largest contributor towards the total attraction (*ca*. 67%–75%), implying their covalent nature. In both Ng–N and Si–Ng bonds, ΔE_disp_ is found to be less important, as it contributes the least.

## 3. Experimental Section

The geometry optimization and the frequency calculation are performed at several levels, *viz*. ωB97X-D/def2-QZVPPD [87,88], MP2/def2-QZVPPD [89] and CCSD(T)/def2-TZVP [90], to ensure that the results obtained are not an artifact of the calculation at a particular level. For the core electrons of Xe and Rn, a quasi-relativistic pseudopotential is used [91]. At both the ωB97X-D/def2-QZVPPD and MP2/def2-QZVPPD levels, H_3_SiNgNSi and HSiNgNSi (Ng = Ar–Rn) are found to be minima on the potential energy surface (PES). However, at the CCSD(T)/def2-TZVP level, the calculations for Ar and Kr inserted analogues do not converge; rather, the compounds dissociate into two fragments during optimization. Hence, we exclude those systems from the discussion. The occurrence of only one imaginary frequency with a mode, which leads to the desired products, implies the transition states (TSs) corresponding to the dissociations of H_3_SiNgNSi and HSiNgNSi into Ng and H_3_SiNSi or HSiNSi. All of these computations are performed by using the Gaussian 09 program package [92]. The atoms-in-molecules (AIM) analysis [71] is carried out by using Multiwfn software [93] at the MP2/def2-QZVPPD/WTBS level. All of electron WTBS [94,95] basis set is used for Xe and Rn.

The energy decomposition analysis (EDA) [72,73,74,75] is performed at the revPBE-D3/TZ2P//MP2/def2-QZVPPD [96,97,98] level using the ADF (2013.01) program package [99]. Scalar relativistic effects are considered for the heavier atoms using the zeroth-order regular approximation (ZORA) [100,101,102].

## 4. Conclusions

H_3_SiNgNSi and HSiNgNSi (Ng = Xe, Rn) could be considered as metastable compounds. The 2-B dissociation pathways producing Ng and H_3_SiNSi or HSiNSi are found to be highly exergonic in nature at room temperature. However, they are found to be kinetically stable along the same dissociation channel due to their activation free energy barriers of 19.3 and 23.3 kcal/mol for H_3_SiXeNSi and H_3_SiRnNSi, respectively, and 9.2 and 12.8 kcal/mol for HSiXeNSi and HSiRnNSi, respectively. The Rn analogues have thermochemical stability with respect to all other possible dissociation channels. However, for H_3_SiXeNSi, another 3-B dissociation channel producing H_3_Si, Xe and NSi is slightly exergonic in nature at 298 K, but at a slightly low temperature (250 K), it turns out to be endergonic in nature. On the other hand, for HSiXeNSi one 2-B (HSiNg and NSi) and 3-B (HSi, Ng and NSi) dissociation paths are slightly exergonic in nature at room temperature. At low temperature (around 150–180 K), they become endergonic. The rest of the dissociation paths are not feasible. According to the argument of Hu *et al.* [77], H_3_SiNgNSi could be stable enough to be detected at the 250–300 K temperature range, whereas HSiNgNSi needs a lower temperature (150–200 K) to be detected. The NPA charge suggests that they could be best represented as (H_3_SiNg)^+^(NSi)^−^ and (HSiNg)^+^(NSi)^−^. Consequently, the WBI values for the Ng–N bonds are found to be quite low (~0.2), whereas the same for the Ng–Si bonds are quite large (~0.65), signifying the covalent nature of the bond. Large ELF values (~0.8) and negative values of ∇^2^ρ(**r_c_**) at the Si–Ng bond critical points further imply its covalent character. As expected from the ionic nature of Ng–N bonds, the maximum contribution in the total attraction energy comes from the ΔE_elstat_ (*ca*. 66%–68%). On the other hand, ΔE_orb_ is the main contributing term (*ca*. 67%–75%) in the total attraction energy of Si–Ng bonds, showing their covalent nature.

## Supplementary Materials

Supplementary materials can be found at http://www.mdpi.com/1422-0067/16/03/6402/s1.
