# Supplementary material for: Exploring the Nature of Silicon-Noble Gas Bonds in H3SiNgNSi and HSiNgNSi Compounds (Ng = Xe, Rn)
Source: Int J Mol Sci. 2015 Mar 19;16(3):6402–18. doi: 10.3390/ijms16036402 (PMC4394539; doi:10.3390/ijms16036402)
Supplement: Supplementary file 1 [file ijms-16-06402-s001.pdf]

Supplementary Information

Table S1. The geometrical parameters (in Å and degrees) of the optimized geometries of $H_3SiNgNSi$ and HSiNgNSi compounds (both minimum energy structures and transition states) studied at the *ω*B97X-D/def2-QZVPPD and CCSD(T)/def2-TZVP levels.

Nature of Stationary Points	Compounds	$r_{H–Si}$	$r_{Si–Ng}$	$r_{Ng–N}$	$r_{N–Si}$	<H–Si–Ng	<Si–Ng–N	<Ng–N–Si
ωB97X-D	$H_3SiXeNSi$	1.475	2.586	2.353	1.561	107.4	180.0	180.0
Minimum	$H_3SiRnNSi$	1.476	2.692	2.398	1.560	108.2	180.0	180.0
Energy	HSiXeNSi	1.516	2.634	2.409	1.562	90.0	180.0	179.1
	HSiRnNSi	1.517	2.739	2.452	1.562	90.2	179.8	179.9
CCSD(T)	$H_3SiXeNSi$	1.478	2.637	2.365	1.585	107.2	180.0	180.0
Minimum	$H_3SiRnNSi$	1.479	2.727	2.404	1.583	108.0	180.0	180.0
Energy	HSiXeNSi	1.517	2.719	2.414	1.586	89.5	179.9	179.5
	HSiRnNSi	1.517	2.796	2.448	1.585	89.9	180.0	180.0

Table S2. The geometrical parameters (in Å and degrees) of the optimized geometries of H_3SiNSi and HSiNSi compounds obtained at the MP2/def2-QZVPPD and ωB97X-D/ def2-QZVPPD levels.

Levels	Compounds	$r_{H–Si}$	$r_{Si–N}$	$r_{N–Si}$	<HSiN	<SiNSi
MP2	H_3SiNSi	1.475	1.723	1.575	110.0	180.0
	HSiNSi	1.519	1.724	1.589	96.6	171.4
ωB97X-D	H_3SiNSi	1.482	1.708	1.554	110.6	180.0
	HSiNSi	1.531	1.705	1.567	97.3	171.6

Table S3. Free energy change (ΔG, kcal/mol) at 298 K for different dissociation channels of $H_3SiNgNSi$ and HSiNgNSi compounds at the MP2/def2-QZVPPD level.

Processes	ΔG		Processes	ΔG	
	Xe	Rn		Xe	Rn
$H_3SiNgNSi \rightarrow H_3SiNg^+ + NSi^-$	209.0	110.5	$HSiNgNSi \rightarrow HSiNg^+ + NSi^-$	204.0	105.0
$H_3SiNgNSi \rightarrow H_3Si^- + NgNSi^+$	286.0	181.3	$HSiNgNSi \rightarrow HSiNg + NSi$	129.1	32.9
$H_3SiNgNSi \rightarrow H_3Si^- + NSiNg^+$	312.7	209.2	$HSiNgNSi \rightarrow HSi^- + NSiNg^+$	308.8	204.7
$H_3SiNgNSi \rightarrow H_3SiNSi + Ng$	−6.7	−101.2	$HSiNgNSi \rightarrow HSi^- + NgNSi^+$	282.1	176.9
$H_3SiNgNSi \rightarrow H_3Si + Ng + NSi$	131.8	37.3	$HSiNgNSi \rightarrow HSiNSi + Ng$	−7.0	−102.1
$H_3SiNgNSi \rightarrow H_3Si^+ + Ng + NSi^-$	226.4	131.8	$HSiNgNSi \rightarrow HSi + Ng + NSi$	127.5	32.4
$H_3SiNgNSi \rightarrow H_3Si^- + Ng + NSi^+$	337.5	242.9	$HSiNgNSi \rightarrow HSi^+ + Ng + NSi^-$	217.4	122.3
$H_3SiNgNSi \rightarrow H_2Si + NgH + NSi$	196.6	102.0	$HSiNgNSi \rightarrow HSi^- + Ng + NSi^+$	333.5	238.4
$H_3SiNgNSi \rightarrow H_2Si^+ + NgH + NSi^-$	310.1	215.4	$HSiNgNSi \rightarrow Si + NgH + NSi$	220.1	124.9
$H_3SiNgNSi \rightarrow H_2Si^- + NgH + NSi^+$	406.4	311.7	$HSiNgNSi \rightarrow Si + NgH^+ + NSi^-$	329.0	227.0
$H_3SiNgNSi \rightarrow H_2Si + NgH^+ + NSi^-$	305.5	204.1	$\Delta G^{‡a}$	10.6	13.5
$H_3SiNgNSi \rightarrow H_2Si^- + NgH^+ + NSi$	372.9	271.4	–	–	–
$H_3SiNgNSi \rightarrow HSi + HNgH + NSi$	286.0	181.5	–	–	–
$H_3SiNgNSi \rightarrow HSi^+ + HNgH + NSi^-$	375.9	271.4	–	–	–
$H_3SiNgNSi \rightarrow HSi^- + HNgH + NSi^+$	492.1	387.5	–	–	–
$\Delta G^{‡a}$	19.1	22.9	–	–	–

$\Delta G^{‡a}$ is the activation free energy barrier for the processes, $H_3SiNgNSi \rightarrow H_3SiNSi + Ng$ and $HSiNgNSi \rightarrow HSiNSi + Ng$.

Table S4. Zero point energy-corrected dissociation energy (D_0, kcal/mol) and dissociation enthalpy at 298 K (ΔH, kcal/mol) for different dissociation channels of $H_3SiNgNSi$ and HSiNgNSi compounds at the MP2/def2-QZVPPD level.

**Processes**	**D_0**		**ΔH**		**Processes**	**D_0**		**ΔH**	
	**Xe**	**Rn**	**Xe**	**Rn**		**Xe**	**Rn**	**Xe**	**Rn**
$H_3SiNgNSi \rightarrow H_3SiNg^+ + NSi^-$	217.7	119.3	217.5	119.1	$HSiNgNSi \rightarrow HSiNg^+ + NSi^-$	212.2	113.4	212.0	113.1
$H_3SiNgNSi \rightarrow H_3Si^- + NgNSi^+$	294.4	189.9	294.7	190.0	$HSiNgNSi \rightarrow HSiNg + NSi$	138.5	42.5	138.4	42.4
$H_3SiNgNSi \rightarrow H_3Si^- + NSiNg^+$	320.3	216.9	319.9	216.5	$HSiNgNSi \rightarrow HSi^- + NSiNg^+$	314.8	211.0	314.3	210.5
$H_3SiNgNSi \rightarrow H_3SiNSi + Ng$	−0.6	−95.1	−0.9	−95.4	$HSiNgNSi \rightarrow HSi^- + NgNSi^+$	288.9	183.9	289.0	184.0
$H_3SiNgNSi \rightarrow H_3Si + Ng + NSi$	147.8	53.3	148.5	54.0	$HSiNgNSi \rightarrow HSiNSi + Ng$	−1.5	−96.3	−1.7	−96.6
$H_3SiNgNSi \rightarrow H_3Si^+ + Ng + NSi^-$	241.1	146.6	241.8	147.3	$HSiNgNSi \rightarrow HSi + Ng + NSi$	141.3	46.4	141.8	46.9
$H_3SiNgNSi \rightarrow H_3Si^- + Ng + NSi^+$	353.4	258.9	354.1	259.6	$HSiNgNSi \rightarrow HSi^+ + Ng + NSi^-$	230.4	135.5	231.0	136.1
$H_3SiNgNSi \rightarrow H_2Si + NgH + NSi$	216.3	121.8	218.1	123.5	$HSiNgNSi \rightarrow HSi^- + Ng + NSi^+$	347.9	253.0	348.5	253.6
$H_3SiNgNSi \rightarrow H_2Si^+ + NgH + NSi^-$	329.8	235.2	331.6	237.0	$HSiNgNSi \rightarrow Si + NgH + NSi$	235.8	140.9	236.9	142.0
$H_3SiNgNSi \rightarrow H_2Si^- + NgH + NSi^+$	426.9	332.4	428.7	334.1	$HSiNgNSi \rightarrow Si + NgH^+ + NSi^-$	342.1	240.4	342.6	240.9
$H_3SiNgNSi \rightarrow H_2Si + NgH^+ + NSi^-$	322.6	221.2	323.9	222.5	$\Delta E^‡$	10.5	13.6	–	–
$H_3SiNgNSi \rightarrow H_2Si^- + NgH^+ + NSi$	390.7	289.4	392.0	290.6	–	–	–	–	–
$H_3SiNgNSi \rightarrow HSi + HNgH + NSi$	302.9	189.5	304.0	199.5	–	–	–	–	–
$H_3SiNgNSi \rightarrow HSi^+ + HNgH + NSi^-$	392.0	287.6	393.1	288.7	–	–	–	–	–
$H_3SiNgNSi \rightarrow HSi^- + HNgH + NSi^+$	509.5	405.1	510.6	406.2	–	–	–	–	–
$\Delta E^‡$	19.7	23.7	–	–	–	–	–	–	–

$\Delta E^‡$ is the activation energy barrier for the processes, $H_3SiNgNSi \rightarrow H_3SiNSi + Ng$ and $HSiNgNSi \rightarrow HSiNSi + Ng$.

Table S5. Zero point energy corrected dissociation energy (D_0, kcal/mol) and dissociation enthalpy at 298 K (ΔH, kcal/mol) for different dissociation channels of $H_3SiNgNSi$ and HSiNgNSi compounds at the ωB97X-D/def2-QZVPPD level.

Processes	D_0		ΔH		Processes	D_0		ΔH	
	Xe	Rn	Xe	Rn		Xe	Rn	Xe	Rn
$H_3SiNgNSi \rightarrow H_3SiNg^+ + NSi^-$	114.4	119.9	114.3	119.8	$HSiNgNSi \rightarrow HSiNg^+ + NSi^-$	107.9	112.7	107.7	112.5
$H_3SiNgNSi \rightarrow H_3Si^- + NgNSi^+$	174.0	175.0	174.4	175.4	$HSiNgNSi \rightarrow HSiNg + NSi$	6.6	14.1	6.7	14.2
$H_3SiNgNSi \rightarrow H_3SiNSi + Ng$	−112.9	−104.1	−113.2	−104.3	$HSiNgNSi \rightarrow HSi^- + NgNSi^+$	171.1	171.4	171.2	171.6
$H_3SiNgNSi \rightarrow H_3Si + Ng + NSi$	16.2	25.1	17.0	25.9	$HSiNgNSi \rightarrow HSiNSi + Ng$	−115.1	−106.8	−115.3	−107.1
$H_3SiNgNSi \rightarrow H_3Si^+ + Ng + NSi^-$	137.1	146.0	137.9	146.8	$HSiNgNSi \rightarrow HSi + Ng + NSi$	8.6	16.9	9.2	17.5
$H_3SiNgNSi \rightarrow H_3Si^- + Ng + NSi^+$	217.9	226.8	218.7	227.6	$HSiNgNSi \rightarrow HSi^+ + Ng + NSi^-$	126.5	134.7	127.1	135.3
$H_3SiNgNSi \rightarrow H_2Si + NgH + NSi$	85.2	94.1	87.1	95.9	$HSiNgNSi \rightarrow HSi^- + Ng + NSi^+$	214.9	223.2	215.6	223.8
$H_3SiNgNSi \rightarrow H_2Si^+ + NgH + NSi^-$	227.3	236.1	229.2	238.0	$HSiNgNSi \rightarrow Si + NgH + NSi$	104.7	113.0	105.9	114.1
$H_3SiNgNSi \rightarrow H_2Si^- + NgH + NSi^+$	293.5	302.4	295.4	304.3	$HSiNgNSi \rightarrow Si + NgH^+ + NSi^-$	233.9	235.7	234.5	236.3
$H_3SiNgNSi \rightarrow H_2Si + NgH^+ + NSi^-$	214.3	216.7	215.7	218.0	$\Delta E^‡$	9.5	12.8	–	–
$H_3SiNgNSi \rightarrow H_2Si^- + NgH^+ + NSi$	255.2	257.6	256.5	258.9	–	–	–	–	–
$H_3SiNgNSi \rightarrow HSi + HNgH + NSi$	166.7	167.2	167.8	168.3	–	–	–	–	–
$H_3SiNgNSi \rightarrow HSi^+ + HNgH + NSi^-$	284.6	285.0	285.7	286.2	–	–	–	–	–
$H_3SiNgNSi \rightarrow HSi^- + HNgH + NSi^+$	373.0	373.5	374.1	374.6	–	–	–	–	–
$\Delta E^‡$	21.2	25.1	–	–	–	–	–	–	–

$\Delta E^‡$ is the activation energy barrier for the processes, $H_3SiNgNSi \rightarrow H_3SiNSi + Ng$ and $HSiNgNSi \rightarrow HSiNSi + Ng$.
